# Immune thrombocytopenia relapse post covid-19 vaccine in young male patient

**DOI:** 10.1016/j.idcr.2021.e01344

**Published:** 2021-11-17

**Authors:** Hana Qasim, Elrazi Ali, Mohamad A. Yassin

**Affiliations:** a Department of Hematology and Medical Oncology, Hamad Medical Corporation, Doha, Qatar; b Internal Medicine Department, Hamad Medical Corporation, Doha, Qatar

**Keywords:** ITP, SARS-CoV-2, Pfizer vaccine, Relapse

## Abstract

Immune thrombocytopenic purpura (ITP) is a blood disorder in which antibodies coating platelets cause platelets destruction in the spleen with resultant low platelets count and an increased tendency for bleeding. Coronavirus disease 2019 (COVID-19) is an illness caused by SARS-COV2; it was first identified in December/2019; though it mainly affects the respiratory system, multisystemic complications are identified. Several ITP cases post mRNA SARS-CoV-2 vaccines were reported, and different pathophysiology theories about the underlying pathophysiology were discussed, but only a few ITP relapse cases have been reported so far. We present a 28-year-old Asian male, a known patient of ITP and in partial remission for eighteen months, who presented to the emergency department with ITP relapse (platelets count of 1 × 10^3 /µL), four days after receiving the second dose of Pfizer SARS-CoV-2 vaccine, which required treatment with intravenous immunoglobulins and dexamethasone. We further discuss the preferred approach in ITP patients who are willing to receive the COVID-19 vaccine.

## Introduction

Immune thrombocytopenic purpura (ITP) is a syndrome in which platelets become coated with autoantibodies to platelet membrane antigens. In patients with ITP, the mononuclear macrophage system of the spleen is responsible for removing platelets with incomplete compensation from the bone marrow. This leads to a decrease in circulating platelets count, which is proved by prompt improvement of patients after splenectomy [1]. Usually, patients present with bruising and mucosal bleeding, with a minority of cases developing major bleeding complications such as intracranial hemorrhage [2]. COVID-19/SARS-CoV-2 infection causes various systemic complications, including acute kidney injury, liver failure, and dermatological manifestation [3], [4], [5]. Complications and mortality are high in patients with multiple comorbidities, including diabetes and hypertension [6], [7]. Additionally, hematological complications are not uncommon in patients with SARS-CoV-2 infection; they include hypercoagulability and subsequent venous thromboembolism, disseminated intravascular coagulation, thrombocytopenia [8], and thrombotic thrombocytopenic purpura [9]. Such complications are more prevalent in more severe cases of infection. Cases of ITP and ITP relapse have been reported in people who received mRNA SARS-CoV-2 vaccine, as well [10], [11], most reported cases present with petechia, bruising, and mucosal bleeding in the first few weeks after receiving the vaccine; nevertheless, the causal relationship is not yet proved.

## Case presentation

We report a 28-year-old male patient with a past medical history of ITP, diagnosed in September/2019. At that time patient presented with an influenza B upper respiratory tract infection. He had no petechial rash, gum bleeding, epistaxis, hematemesis, hematuria, or hematochezia. Blood tests at that time showed a platelet count of 3 × 10^3 /µL (150–400 × 10^3/µL), normal hemoglobin, and normal white blood cell count (WBC). Hepatitis B surface antigen, hepatitis C antibodies, helicobacter pylori stool antigen, and human immunodeficiency virus (HIV) antigen/antibodies were negative. Autoimmune workup was negative for Antineutrophil cytoplasmic antibodies (ANCA), antinuclear antibodies, anti-double-stranded DNA antibodies, ant-Jo, anti-RNP, and anti-La antibodies, while anti-RO antibodies came positive.

Ultrasound abdomen was done to assess for hepatosplenomegaly, and it came normal.

The hematology team evaluated the patient, and their impression was ITP secondary to viral infection. The patient received five days course of 0.5 mg/kg Intravenous immune globulin (IVIG) and four days of intravenous (IV)dexamethasone 40 mg daily. By the end of the treatment, the patient's platelets count went up to 201 × 10^3 /µL. He was discharged home on prednisolone 1 mg/kg, tapered over four weeks. The patient was followed in the hematology clinic in November/2019, he had no petechia or mucosal bleeding, and the repeated platelets count was 45 × 10^3 /µL. He was doing well with no signs of petechia or mucosal bleeding since then. On the 1st of April 2021, the patient received the second dose of Pfizer SARS-CoV-2 vaccine. On the 5th of April 2021, he presented to the emergency department with epistaxis and petechiae for two days. He had no gum bleeding, rectal bleeding, hematemesis, hematuria, or fever. Physical examination showed normal vital signs, petechial rash on upper and lower extremities, and no hepatosplenomegaly on abdominal examination. Lab tests showed platelets count of 1 × 10^3 /µL, WBC count of 9.4 × 10^3 /µL (normal level: 4–10 × 10^3 /µL), Hemoglobin of 14.9 gm/dl (normal level: 12–15 gm/dl), and normal kidney and liver function. He was started on IVIG 0.5 mg/kg and dexamethasone 40 mg IV daily for four days. After the treatment course, platelets count improved to 60 × 10^3 /µL, and he was discharged home on prednisolone 1 mg/kg tapering regimen over one month.

## Discussion

There has been increased recognition of post mRNA SARS-CoV-2 vaccines thrombocytopenia recently. Some of these cases are for patients with previous normal platelet count [11] and others for patients with previous chronic stable thrombocytopenia [12]. The aim of medical treatment for immune thrombocytopenia (ITP) is to increase the platelet count to a safe level while awaiting spontaneous or treatment-induced remission. Our patient has a history of ITP, with no symptoms for 18 months; his ITP flare was temporally related to the COVID-19 vaccine. We propose that the COVID-19 vaccine induced a relapse of his previously stable ITP. Multiple mechanisms have been discussed for COVID-19 infection-induced thrombocytopenia, including platelets destruction via molecular mimicry, cryptic antigen expression, and the decrease in platelets production in the bone marrow due to dysfunctional marrow microenvironment [10]. It is not clear whether the underlying mechanism of ITP post the COVID19 vaccine is the same as the previously described mechanisms. Post vaccine ITP had been reported after many vaccines previously, including recombinant zoster vaccine [13], varicella vaccine [14], and in a large study in children who received MMR vaccine [15]. The mechanisms of vaccines induced ITP were discussed before, like T cell dysregulation, increased production of the pro-inflammatory cytokine, and enhancement of macrophage-mediated clearance, which might apply to the COVID-19 vaccine as well [16]. With the significant number of people who have received covid vaccine worldwide, and the rarity of the associated ITP cases that were reported so far, the causal relationship between the covid vaccine and ITP is still questionable. Even if proven association with a few numbers of patients, the prompt improvement of patients after receiving the standard management of ITP, and the significant role of covid vaccine to contain this pandemic, the benefits of this vaccine still by far outweigh the potential risks. Nevertheless, for people who are known to have thrombocytopenia, obtaining complete blood count before receiving the mRNA SARS-CoV-2 vaccines and weekly follow up for the first 3–4 weeks after that might be a reasonable approach for early recognition and management of possible ITP relapse.

## Authors contributions

**Hana Qasim**: literature review, Manuscript writing, Conception, acquisition of data, Drafting the manuscript, revising the manuscript critically for important intellectual content, corresponding author. **Elrazi Ali**: Manuscript writing and editing. **Mohamad A Yassin**: literature review, Manuscript writing, Drafting the manuscript, revising the manuscript critically for important intellectual content. Approval of the version of the manuscript by all authors.

## Funding

Open Access funding provided by the Qatar National Library, Qatar.

## Ethical approval

Private information will not be published, ethical approval is not required.

## Consent

Informed consent was taken.

## Statement of Ethics

The case is approved by Hamad Medical Corporation medical research center.
